# Pumping the brakes: A noncanonical RNA-binding domain in FMRP stalls elongating ribosomes

**DOI:** 10.1016/j.jbc.2022.102773

**Published:** 2022-12-05

**Authors:** Sara K. Young-Baird

**Affiliations:** Department of Biochemistry and Molecular Biology, F. Edward Hébert School of Medicine, Uniformed Services University, Bethesda, Maryland, USA

**Keywords:** RNA-binding protein, protein synthesis, translational control, FMRP, fragile X mental retardation protein, fragile X syndrome, CTD, C-terminal domain, FMRP, fragile X mental retardation protein, FXS, fragile X syndrome, G4, G-quadraplex, ncRBD, noncanonical RNA-binding domain

## Abstract

Loss of function of the RNA-binding protein FMRP causes fragile X syndrome, the most common inherited form of intellectual disability and autism spectrum disorders. FMRP is suggested to modulate synaptic plasticity by regulating the synthesis of proteins involved in neuronal and synaptic function; however, the mechanism underlying FMRP mRNA targeting specificity remains unclear. Intriguing recent work published in *JBC* by Scarpitti and colleagues identifies and characterizes a noncanonical RNA-binding domain that is required for FMRP-mediated translation regulation, shedding light on FMRP function.

Fragile X syndrome (FXS), caused by loss of function of the fragile X mental retardation protein (FMRP), is the most common inherited form of intellectual disability and largest single genetic driver of autism (1). The majority of FXS cases result from expansion of a CGG trinucleotide repeat in the *fragile X mental retardation 1* gene, leading to transcriptional silencing of the locus and diminished expression of FMRP (2). This link between FMRP activity and FXS presentation has prompted extensive research into the functions of FMRP with an eye toward pharmacological intervention for the disease.

Through the work of multiple groups, FMRP has been characterized as an RNA-binding protein with roles in the regulation of mRNA splicing, stability, localization, and translation (1, 3). Consistent with patient symptomatic presentation, FMRP is highly expressed in the developing brain and appears to regulate neuron function and synaptic plasticity by binding transcripts encoding pre- and postsynaptic, as well as autism-associated, proteins (4, 5, 6). However, the mechanism underlying FMRP mRNA substrate selection and how FMRP enacts its various molecular activities has been unclear and may be context dependent. These complexities in FMRP function have made therapeutic development for FXS difficult, and treatments have thus far been geared toward the management of disease symptoms (7).

New work by Scarpitti *et al.* provides a fresh take on FMRP function, focusing on the ability of FMRP to bind mRNA and directly regulate translation (8). In their initial experiments, the authors tested a leading model that FMRP inhibits translation elongation after being recruited to target transcripts by binding G-quadraplex (G4) RNA structures within the mRNA protein coding sequence (1). Key to these studies were elegantly designed NanoLuc (nLuc) luciferase translational reporters that either lacked or contained RNA G4 structures within the coding sequence (8). If G4s are in fact required for mRNA recruitment and translational inhibition by FMRP, the authors postulated that incubation of G4-containing luciferase reporters with affinity-purified human FMRP would result in decreased nLuc activity in *in vitro* translation assays. Intriguingly, FMRP repressed translation of both the control and G4-containing mRNAs, suggesting that FMRP actually inhibits translation independent of mRNA G4 structures.

If G4s do not play a role in FMRP recruitment to mRNAs, how then does FMRP select and inhibit the translation of its target transcripts? Scarpitti and colleagues address this question by dissecting the domain structure of FMRP. FMRP contains three canonical RNA-binding domains (KH0, KH1, and KH2), an arginine-glycine-rich (RGG) box motif, and a C-terminal domain (CTD) (1). Patients with FXS have been identified with mutations in the KH1, KH2, and RGG domains emphasizing the significance of these domains for FMRP function and suggesting that multiple regions of FMRP may play a role in its RNA-binding ability. To define the domain(s) that are essential for FMRP-mediated translation regulation, the authors purified wild-type and mutant FMRP harboring different FXS patient mutations, as well as truncated recombinant proteins containing the RGG box motif and CTD regions either alone or together (8). Importantly, one of the FXS patient mutations utilized in this analysis introduces a premature termination codon in the sequence encoding the RGG motif, resulting in a protein lacking most of the RGG and the entire CTD region. Unlike wild-type FMRP, this FXS mutant protein did not repress *in vitro* translation of an nLuc mRNA reporter, indicating that the RGG and CTD regions are essential for translational repression by FMRP. Corroborating this idea, robust translational repression was observed with a truncated FMRP protein containing the RGG motif and CTD, but neither the RGG nor CTD alone was sufficient to inhibit translation. With the RGG and CTD defined as the domains required for FMRP-mediated translational repression, the authors next sought to determine the RNA binding capability of this region. For this analysis, FAM-labeled homopolymeric RNAs and truncated recombinant proteins containing the RGG and CTD were utilized in electrophoretic mobility shift assays and fluorescence polarization experiments. Interestingly, the RGG plus CTD region robustly bound homopolymeric A, C, U, and G RNA sequences, suggesting that the RGG and CTD together form a noncanonical RNA-binding domain (ncRBD) and that FMRP mRNA binding may be more promiscuous than previously appreciated.

FMRP’s role in translational regulation is an unresolved mechanistic question in the field. Work by several groups has suggested that FMRP may inhibit translation at the steps of both initiation and elongation (5, 6, 9, 10). To address the precise timing of FMRP translational inhibition in their assays, Scarpitti *et al.* developed multiple unique strategies, only one of which is discussed here. In an insightful experiment geared to assess translation elongation, the authors tested FMRP’s ability to promote the accumulation of puromycin-resistant ribosomes on their nLuc reporter mRNA (8). Puromycin is an acyl tRNA analog that causes the release of nascent polypeptides from actively elongating 80S ribosomes. However, slowly elongating, stalled ribosomes exist in a rotated state that is resistant to puromycin treatment. Utilizing *in vitro* translation reactions, the authors found that puromycin-resistant ribosomes accumulated on the reporter mRNA in the presence of wild-type FMRP but not in reactions containing FMRP lacking the RGG and CTD regions, which were required for FMRP-mediated translational inhibition as described above. Together, the data from these meticulous and complimentary experiments bolster evidence for a model in which FMRP utilizes an ncRBD made up of the RGG and CTD to bind mRNAs and stall elongating ribosomes (Fig. 1).

**Figure 1:**
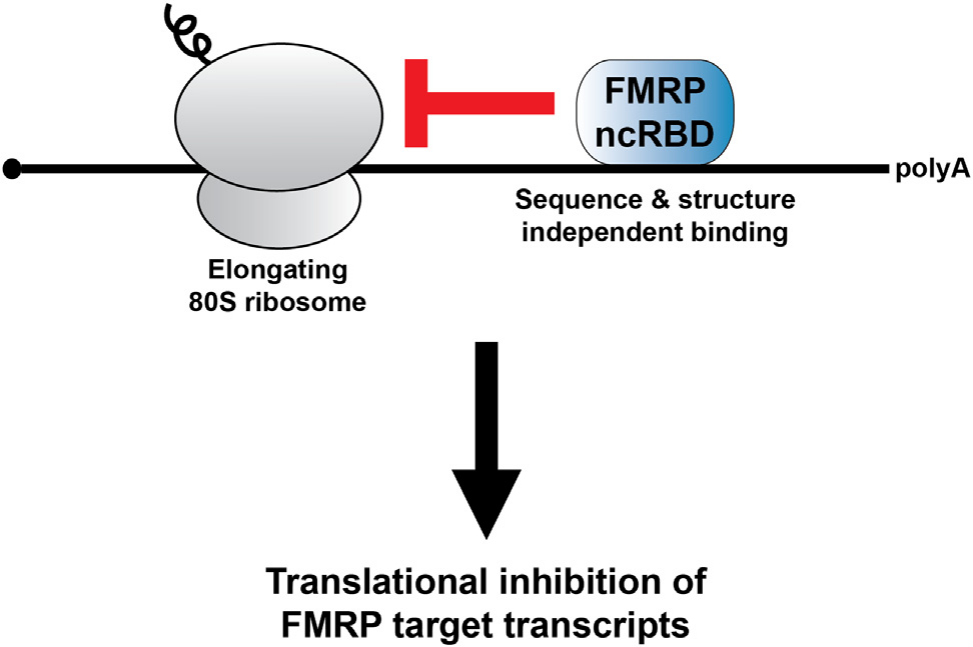
**Model of FMRP-mediated translational inhibition.** A promiscuous noncanonical RNA-binding domain (ncRBD) in FMRP facilitates binding to target transcripts and subsequent inhibition of translation elongation.

While this study clearly advances our understanding of FMRP function, it also reemphasizes the longstanding question of how FMRP selects its mRNA substrates. If FMRP binding is promiscuous, could FMRP regulate translation of neuronal mRNAs primarily based on the local concentration of the protein and not mRNA sequence? Alternatively, perhaps the function of FMRP in neurons is dictated by coordination of the various FMRP RNA-binding domains to select specific mRNA sequences or structures that could not be identified in the authors’ detailed analysis of the newly identified FMRP ncRBD. A clearer picture of the RNA-binding modality of FMRP, and its potential context dependence, will be critical for informed therapeutic approaches targeting the molecular basis of FXS etiology.

## Conflicts of interest

The author declares that they have no conflicts of interest with the contents of this article.
